# Trends in the Hospital Care of Neonates With Hypoplastic Left Heart Syndrome in the United States

**DOI:** 10.1016/j.atssr.2022.11.013

**Published:** 2022-11-25

**Authors:** Jason W. Greenberg, Farhan Zafar, Alia Dani, David S. Winlaw, Haleh C. Heydarian, Samuel P. Hanke, David S. Cooper, James S. Tweddell, David L.S. Morales

**Affiliations:** 1 The Heart Institute, Cincinnati Children’s Hospital Medical Center, University of Cincinnati College of Medicine, Cincinnati, Ohio

## Abstract

**Background:**

Surgical outcomes of first-stage palliation for hypoplastic left heart syndrome (HLHS) are vastly improved from prior eras and are well described, but rates and determinants of nonsurgical management are understudied, particularly on the national scale.

**Methods:**

The Pediatric Health Information System database was used to identify all index neonatal HLHS admissions between 2015 and 2021 in the United States. Surgical palliation included cardiac surgery and transplantation during the index admission. Multivariable binary logistic regression was used to identify factors independently predictive of nonsurgical management, represented by odds ratio <1.0.

**Results:**

In total, 3902 HLHS neonatal admissions to 48 tertiary care centers occurred. Eighteen percent (n = 710) of neonates did not receive surgical palliation. Ninety-seven percent of nonsurgical patients died in the hospital or were discharged to comfort care, whereas 84% of surgically palliated neonates survived to discharge. Nonsurgical patients had greater rates of prematurity, birth weight <2.5 kg, female sex, and chromosomal abnormality (all *P* < .01). Factors independently associated with nonoperative management included admission to a low-volume surgical program (odds ratio, 0.2 [95% CI, 0.15-0.26]), birth weight <2.5 kg (0.30 [0.24-0.37]), chromosomal abnormality (0.55 [0.43-0.71]), and non-White race/ethnicity (0.68 [0.56-0.82]).

**Conclusions:**

In the contemporary era of neonatal HLHS management, nearly one-fifth of neonates do not undergo surgical palliation in the United States. Additional work can outline the precise reasons underlying such high rates of nonoperative management in hopes of ensuring equitable care for all children with HLHS.

**In Short**

▪ In the modern era of hypoplastic left heart syndrome (HLHS) management, nearly 20% of HLHS neonates admitted to tertiary care centers with cardiac surgery capabilities do not receive surgical palliation.
▪ Contemporary trends in operative management suggest that access to surgical care is not equal; neonates with certain characteristics (non-White, chromosomal abnormality) and those admitted to low-volume programs are less likely to have a surgical procedure.
▪ These findings can help inform clinical decision-making and highlight additional avenues for quality improvement.

With an incidence of approximately 2 cases per 10,000 births in the United States, hypoplastic left heart syndrome (HLHS) is the most common and severe form of single-ventricle congenital heart disease.^1^^,^^2^ The diagnosis and management of HLHS have improved significantly in recent decades. Presently, most patients are diagnosed in utero, allowing management decision-making to ensue even before birth.^3^^,^^4^ When surgical palliation is elected, the first-stage operation is typically performed during the first week of life. Several recent single-center reports have cited survival to discharge or 1 month of age in 90% of surgical patients.^3^^,^^5^^,^^6^ Yet, trends in the care chosen for neonates with HLHS admitted to the hospital are understudied on the national level. Knowledge of such phenomena can help inform clinical decision-making and guide future research and quality improvement efforts.

## Patients and Methods

Study approval was granted through waiver of informed consent by the Cincinnati Children’s institutional review board (#2018-6837). The authors conducted a retrospective, diagnosis-based cohort study to characterize contemporary national trends in rates and determinants of neonatal HLHS surgical palliation, with an emphasis on patient characteristics and program size. Secondary analyses focused on hospital outcomes.

Data were obtained using the Pediatric Health Information System (PHIS), an administrative data set of tertiary care pediatric hospitals in the United States. Unlike similar outcomes-based quality improvement data sets, PHIS has the important advantage of being predicated on hospital admissions and inpatient management, thereby allowing trends in both operative and nonoperative management to be studied.

*International Classification of Diseases, Tenth Revision* (ICD-10) codes were used to identify all patients with a diagnosis of HLHS (Q23.4) admitted to a PHIS-affiliated hospital during the first 30 days of life between October 1, 2015, and July 30, 2021. Analysis was limited to the index neonatal admission, defined as admissions on day 1 of life or transfer from an outside hospital to a PHIS-affiliated tertiary care center for definitive management before day 30 of life. Readmissions during the first 30 days of life were not considered index neonatal admissions.

Surgical palliation was defined as cardiac surgery or heart transplantation during the index neonatal admission (ICD-10 procedure codes in Supplemental Table 1). Patients without an ICD-10 code for cardiac surgery or heart transplantation were considered to have received nonoperative (comfort care) management. All hospitals included in analysis performed at least 1 HLHS surgery or transplant during the study period.

Programs were categorized as low, medium, or high volume, depending on quartile of combined average neonatal HLHS cardiac surgery and heart transplantation during the 5 study years with complete data for the annum (2016-2020). Forty-eight programs were identified: low volume, n = 12 (≤7 HLHS operations annually); medium volume, n = 23; and high volume, n = 13 (≥18 HLHS operations annually). Low birth weight was defined as <2.5 kg, and preterm birth was defined as <37 weeks’ gestation. Chromosomal abnormalities were identified by ICD-10 diagnosis codes Q90 to Q99. Insurance (payer) was categorized as private (health maintenance organizations, preferred provider organizations, self-pay) or public (governmental; Medicaid, Medicare, other state- or federal-based insurance) as designated by PHIS.

Statistical analyses used R (R Foundation for Statistical Computing). Brown-Mood median test was used to compare continuous variables, with Pearson *χ*^2^ test with Yates continuity correction and Fisher exact test (for cell counts <10) used for categorical variables. Multivariable binary logistic regression was performed to identify factors independently associated with surgical palliation (odds ratio >1.0). Backward stepwise elimination of covariates with *P* < .10 was performed to create the final multivariable model (Supplemental Table 2).

## Results

A total of 3902 neonatal HLHS admissions were identified. Most (83%) were born at term and weighed >2.5 kg (82%), were of White race (53%), did not have a chromosomal abnormality (88%), and had public/governmental insurance (54%; Supplemental Table 3).

Differences existed in patient characteristics by program size (Table 1). Patients admitted to low-volume centers had the greatest percentage of low birth weight (25%; *P* < .01 vs medium-volume [18%] and high-volume [17%] centers) and the greatest proportions of public insurance (69%; *P* < .001 vs medium-volume [54%] and high-volume [51%] centers). Patients cared for at high-volume centers had a lower incidence of chromosomal abnormalities (10%) than patients cared for at medium-volume (14%) or low-volume (15%) centers (*P* < .02 vs both).

**Table 1:** Characteristics of Patients by Program Volume

Characteristic	Low Volume (n = 316)	Medium Volume (n = 1714)	High Volume (n = 1872)	*P* Value
Low birth weight	67 (25)	288 (18)	275 (17)	.005
Preterm birth	52 (20)	228 (18)	233 (16)	.161
Sex, male	166 (52)	1008 (59)	1179 (63)	.001
Race/ethnicity				
Asian	4 (1)	39 (2)	21 (1)	.022
Black	46 (15)	280 (16)	201 (11)	<.001
Hispanic	85 (27)	302 (18)	296 (16)	<.001
White	153 (48)	846 (50)	1036 (55)	.004
Chromosomal abnormality	47 (15)	237 (14)	191 (10)	.001
Household income ($1000)	41 (32-49)	39 (33-50)	40 (33-51)	.005
Public/government insurance	69%	54%	51%	<.001

Values are expressed as median (interquartile range) or number (percentage) as appropriate.

Overall, 82% (n = 3192) of HLHS neonates received surgical palliation, with 18% (n = 710) receiving nonoperative management. Most surgical patients underwent cardiac surgery alone (97%; n = 3102), with a minority undergoing up-front heart transplantation (2%; n = 58) or subsequent heart transplantation during the neonatal admission after up-front cardiac surgery (1%; n = 32). Compared with nonsurgical patients, neonates who received cardiac surgery had lower rates of preterm birth, low birth weight, and chromosomal abnormalities (Table 2). Whereas patients of all races and ethnicities were more likely to undergo surgical palliation than not, only 66% of Asian neonates received palliative operations compared with approximately 80% or more among other race/ethnicity categories. Neither median household income nor the proportion of patients with public/governmental insurance was different between the surgical and nonsurgical groups (Table 2).

**Table 2:** Characteristics of Surgical vs Nonsurgical Patients

Characteristic	Surgical Palliation (n = 3192)	No Surgery (n = 710)	*P* Value
Birth weight <2.5 kg	399 (14)	231 (36)	<.001
Preterm birth	332 (14)	181 (33)	<.001
Male sex	1960 (61)	393 (55)	.004
Race/ethnicity			
Asian	42 (66)	22 (34)	.001
Black	426 (81)	101 (19)	.576
Hispanic	540 (79)	143 (21)	.047
White	1738 (85)	315 (15)	<.001
Chromosomal abnormality	335 (11)	140 (20)	<.001
Household income ($1000)	40 (33-51)	41 (32-50)	.601
Public/governmental insurance	1715 (54)	376 (53)	.743

Values are expressed as median (interquartile range) or number (percentage) as appropriate.

Surgical rates differed by program size. At low-volume programs, 56% of patients received surgical palliation compared with 83% at medium-volume and 85% at high-volume programs (*P* < .001 for low- vs medium- and high-volume programs; *P* = .338 for medium- vs high-volume programs). The proportion of patients with chromosomal abnormalities who underwent surgical palliation was 49% at low-volume programs compared with 74% at medium-volume and 71% at high-volume programs (*P* < .01 for low- vs medium- and high-volume programs; *P* = .662 for medium- vs high-volume programs). Median total hospital charges for surgical patients were $845,000 (interquartile range [IQR], $508,000-1,610,000) compared with $41,000 (IQR, $32,000-$50,000) for nonsurgical patients (*P* < .001). At low-volume programs, the median cost of surgical admissions was $1,166,000 (IQR, $539,000-$1,829) compared with $872,000 (IQR, $554,000-$1,624,000) at medium-volume and $804,000 ($482,000-$1,518,000) at high-volume programs (*P* ≤ .001 for low- vs medium- and high-volume programs; *P* = .053 for medium- vs high-volume programs).

Eighty-four percent (n = 2674) of surgically palliated patients were discharged to a rehabilitation facility or to home, with 15% (n = 483) experiencing in-hospital mortality and 1% (n = 17) transferred to another pediatric hospital (with subsequent management unclear). Ninety-seven percent (n = 668) of nonsurgical patients died in the hospital or were discharged to hospice, with 3% (n = 21) transferred to another pediatric hospital. By program volume, postoperative mortality occurred in 9% of surgically palliated patients at low-volume centers compared with 16% at medium-volume and 14% at high-volume centers (*P* = .707).

Factors independently associated with surgical palliation included White ethnicity (odds ratio, 1.47 [95% CI, 1.22-1.78]; *P* < .001) and public/governmental insurance (1.37 [1.13-1.67]; *P* = .001). Factors independently associated with nonsurgical management included admission to a low-volume surgical program (0.20 [0.15-0.26]; *P* < .001), birth weight <2.5 kg (0.30 [0.24-0.37]; *P* < .001), and presence of a chromosomal abnormality (0.55 [0.43-0.71]; *P* < .001).

## Comment

As reported by the Single Ventricle Reconstruction trial and other single-center studies, early and midterm outcomes for neonatal HLHS surgical palliation are vastly improved.^3^^,^^5^, ^6^, ^7^ In this analysis, 84% of neonates who underwent surgical palliation survived to hospital discharge, whereas 97% of those managed nonsurgically died in the hospital or were discharged to comfort care. However, with regard to surgical management, the care of neonates who presented to the hospital with HLHS was, seemingly, *not* equitable.

Across the United States, 18%—nearly 1 in 5—neonates born with HLHS did not receive surgical palliation. Importantly, only 3% of these patients were transferred to another pediatric hospital for care, suggesting that the notion that many nonsurgical patients were simply transferred to another center for surgical procedures incorrect.

Trends emerged differentiating patients who received surgical palliation from those who did not. Although most patients born with the high-risk features of prematurity, low birth weight, and chromosomal abnormalities *did* receive surgical palliation, patients with these characteristics made up a disproportionate percentage of the nonsurgical group, especially in low-volume centers. Low birth weight, chromosomal abnormality, and non-White race/ethnicity were independently associated with nonsurgical management.

The *distribution* of care was also seemingly not equal. Nonsurgical management rates were seemingly disproportionate at smaller programs: 44% of patients admitted to low-volume centers did not receive surgical palliation compared with <20% at medium- and high-volume centers. Patients admitted to low-volume centers were of slightly higher risk, with a greater proportion of patients with low birth weight and chromosomal abnormalities, but those admitted to medium- or high-volume centers were significantly more likely to receive cardiac surgical operations even in the presence of these risk factors. Despite similar postsurgical hospital outcomes across all programs, surgical admissions were costlier at low-volume centers—by nearly $400,000 compared with high-volume centers. Although provocative, these findings require further study. For example, whereas it is against the law to not operate on patients because of the presence of a chromosomal or genetic abnormality, a number of these patients can have concomitant comorbidities that preclude surgical management. However, case mix indices and information about other comorbidities are not available in PHIS; both may not have been fully accounted for in multivariable analysis.

Additional limitations exist. Several important determinants of care, such as social determinants of health, familial preference, impact of perinatal counseling, and consideration for long-term prognosis, could not be assessed. Specifically, access to and rates of pregnancy termination likely influenced regional management trends, but this also could not be studied. In addition, Asian patients received surgery at the lowest rate of any group, but the small number of Asian neonates precluded further meaningful analysis. Like all retrospective database-driven studies, reporting biases must be considered. Only patients admitted to 1 of 48 PHIS-affiliated hospitals were studied. The findings herein are therefore not wholly generalizable to all pediatric hospitals and would benefit from expansion and validation using other sources, such as the Kids’ Inpatient Database, which contains data from >4000 community hospitals across 48 states.

In conclusion, in the modern era of HLHS management, nearly 20% of HLHS neonates admitted to tertiary care pediatric hospitals do not receive surgical palliation. The rate of nonoperative management in this study must be viewed in the context of long-term outcomes, which have historically been suboptimal, but contemporary trends in operative management suggest that access to surgical care is not equal. Additional work is required to elucidate the precise reasons underlying nonoperative management, but the findings of this analysis can help inform clinical decision-making and highlight further avenues for quality improvement while working toward the insurance of equitable care for all patients.
