# Supplementary material for: Trends in the Hospital Care of Neonates With Hypoplastic Left Heart Syndrome in the United States
Source: Ann Thorac Surg Short Rep. 2022 Nov 25;1(1):70–3. doi: 10.1016/j.atssr.2022.11.013 (PMC11708278; doi:10.1016/j.atssr.2022.11.013)
Supplement: Supplementary Materials [file mmc1.docx]

**Supplementary**

| 02QM0ZZ | 021K0KP | 021V0KP | 021R0JD | 021X0ZR | 021Q08B | 021X0ZQ | 021L0KP | 02UW0JZ |
| --- | --- | --- | --- | --- | --- | --- | --- | --- |
| 02UM07Z | 021K0ZP | 021V0ZP | 021P0AA | 03140JN | 021Q09B | 021X08R | 021L0JQ | 02UW0KZ |
| 02UM08Z | 021K0JQ | 021K09Q | 021P0JA | 03140ZN | 021Q0AB | 021X09R | 021L0KR | 02UX07Z |
| 02UM0JZ | 021K0KQ | 021K0AQ | 021P0KA | 03140ZM | 021Q0JB | 021X0AR | 5A1221Z | 02UX08Z |
| 02U507Z | 021K0ZQ | 021K0JQ | 021P0ZA | 03130JM | 021Q0KB | 021X0JR | 02Q53ZZ | 02UX0JZ |
| 02U508Z | 021K0AR | 021K0KQ | 021P0JB | 03130ZM | 021Q0ZB | 021X0KR | 02T50ZZ | 02UX0KZ |
| 02U50JZ | 021K0JR | 021K0ZQ | 021P0JD | 021608P | 021R0JD | 021X0ZR | 02B50ZZ | 02RW07Z |
| 02U50KZ | 021K0KR | 021K0AR | 021W08P | 021609P | 021P0AA | 03140JN | 02550ZZ | 02RW08Z |
| 02Q50ZZ | 021K0ZR | 021K0JR | 021W09P | 02160AP | 021R0JA | 03140ZN | 02163Z7 | 02RW0JZ |
| 02UG08Z | 02BK0ZZ | 021K0KR | 021W0AP | 02160JP | 021R0KA | 03140ZM | 02LR0CT | 02RW0KZ |
| 02UG0JZ | 027K0DZ | 021K0ZR | 021W0JP | 02160KP | 021R0ZA | 03130JM | 02LR0DT | 02RX07Z |
| 02UG0KZ | 027K0ZZ | 021K08P | 021W0KP | 02160ZP | 021P0JB | 03140ZM | 02LR0ZT | 02RX08Z |
| 02QG0ZE | 02UP07Z | 021K09P | 021W0ZP | 021608Q | 021P0JD | 02SP0ZZ | 02VR0CT | 02RX0JZ |
| 02QG0ZZ | 02UP08Z | 021K0AP | 021W08Q | 021609Q | 021W08P | 02SX0ZZ | 02VR0DT | 02RX0KZ |
| 02RG08Z | 02UP0JZ | 021K0JP | 021W09Q | 02160AQ | 021W09P | 02BX0ZZ | 02VR0ZT | 02QW0ZZ |
| 02RG0JZ | 02UP0KZ | 021K0KP | 021W0AQ | 02160JQ | 021W0AP | 02BW0ZX | 02QP0ZZ | 02QX0ZZ |
| 02UJ07Z | 02QP0ZZ | 021K0ZP | 021W0JQ | 02160KQ | 021W0JP | 02BW0ZZ | 02QQ0ZZ | 02UW07Z |
| 02UJ08Z | 02BP0ZZ | 021K09Q | 021W0KQ | 02160ZQ | 021W0KP | 02QW4ZZ | 02QR0ZZ | 02UW08Z |
| 02UJ0JG | 02RP0KZ | 021K0AQ | 021W0ZQ | 021608R | 021W0ZP | 04R007Z | 02UP07Z | 02UW0JZ |
| 02UJ0JZ | 027P0DZ | 021K0JQ | 021W08R | 021609R | 021W08Q | 02RH07Z | 02UP08Z | 02UW0KZ |
| 02UJ0KG | 027P0ZZ | 021K0KQ | 021W09R | 02160AR | 021W09Q | 02RH0JZ | 02UP0JZ | 02UX07Z |
| 02UJ0KZ | 02VR0ZZ | 021K0QZ | 021W0AR | 02160JR | 021W0AQ | 02RH0KZ | 02UP0KZ | 02UX08Z |
| 02QJ0ZG | 02VQ0ZZ | 021K0AR | 021W0JR | 02160KR | 021W0JQ | 02RG08Z | 02UQ07Z | 02UX0JZ |
| 02QJ0ZZ | 02NH0ZZ | 021K0JR | 021W0KR | 02160ZR | 021W0KQ | 02RG0JZ | 02UQ08Z | 02UX0KZ |
| 02RJ08Z | 02BH0ZZ | 021K0KR | 021W0ZR | 06100JP | 021W0ZQ | 02NF0ZZ | 02UQ0JZ | 02RW07Z |
| 02RJ0JZ | 02RH07Z | 021K0QR | 021X08P | 06100KP | 021W08R | 02BF0ZZ | 02UQ0KZ | 02RW08Z |
| 024G072 | 02RH0JZ | 021R09A | 021X09P | 06100ZP | 021W09R | 02RF07Z | 02UR07Z | 02RW0JZ |
| 02UJ0JG | 02RH0KZ | 021R0JA | 021X0AP | 061007Q | 021W0AR | 02RF08Z | 02UR08Z | 02RW0KZ |
| 02UJ0KG | 02RH07Z | 021R0KA | 021X0JP | 06100JQ | 021W0JR | 02RF0KZ | 02UR0JZ | 02RX07Z |
| 02QG0ZE | 02RH0JZ | 021R0JB | 021X0KP | 06100KQ | 021W0KR | 02TF0ZJ | 02UR0KZ | 02RX08Z |
| 02QJ0ZG | 02RH0KZ | 021R0ZB | 021X0ZP | 021R09A | 021W0ZR | 02QF0ZZ | 02RP0KZ | 02RX0JZ |
| 024F0JJ | 02TH0ZZ | 021R0JD | 021X08Q | 021R0JA | 021X08P | 02QF0ZJ | 02RQ07Z | 02RX0KZ |
| 02UK07Z | 021V08Q | 021Q08A | 021X09Q | 021R0KA | 021X09P | 02QF0ZZ | 02RQ0JZ | 02BW0ZX |
| 02UK08Z | 021V09Q | 021Q09A | 021X0AQ | 021R0JB | 021X0AP | 02VP0CZ | 02RQ0KZ | 02BX0ZZ |
| 02UK0JZ | 021V0JQ | 021Q0JA | 021X0JQ | 021R0ZB | 021X0JP | 02VP0DZ | 02RR07Z | 02170ZT |
| 02UK0KZ | 021V0KQ | 021Q0KA | 021X0KQ | 021R0JD | 021X0KP | 02VP0ZZ | 02RR0JZ | 02170ZU |
| 02QK0ZZ | 021V0ZQ | 021Q0ZA | 021X0ZQ | 021Q08A | 021X0ZP | 02VR0CZ | 02RR0KZ | 02170ZS |
| 02NK0ZZ | 021V0AR | 021Q08B | 021X08R | 021Q09A | 021X08Q | 02VR0DZ | 021X0JP |  |
| 021K08P | 021V0JR | 021Q09B | 021X09R | 021Q0AA | 021X09Q | 02VQ0CZ | 02QW0ZZ |  |
| 021K09P | 021V0KR | 021Q0JB | 021X0AR | 021Q0JA | 021X0AQ | 02VQ0DZ | 02QX0ZZ |  |
| 021K0AP | 021V0ZR | 021Q0KB | 021X0JR | 021Q0KA | 021X0JQ | 02VQ0ZZ | 02UW07Z |  |
| 021K0JP | 021V0JP | 021Q0ZB | 021X0KR | 021Q0ZA | 021X0KQ | 021L0JP | 02UW08Z |  |

**Table S1. ICD-10 procedure codes for HLHS surgical palliation and heart transplantation.**

|  | **OR (95% CI)** | **p-value** |
| --- | --- | --- |
| White ethnicity | 1.47 (1.22-1.78) | <0.001 |
| Public/government insurance | 1.37 (1.13-1.67) | 0.001 |
| Chromosomal abnormality | 0.55 (0.43-0.71) | <0.001 |
| Birthweight <2.5 kg | 0.30 (0.24-0.37) | <0.001 |
| Low-volume program | 0.20 (0.15-0.26) | <0.001 |
|  |  | |
| Median household income | *Covariate omitted from final multivariable model for p>0.10 on backward stepwise elimination.* | |
| Male sex |  |  |

**Table S2. Multivariable model of odds of receiving HLHS neonatal surgical palliation.**

Odds ratio (OR) >1.0 indicates positive association with surgical palliation. CI, confidence interval.

| Number of patients | 3,902 |
| --- | --- |
|  |  |
| Gestational age (weeks) | 38 (37–39) |
| Low birthweight | 630 (18%) |
| Preterm birth | 513 (17%) |
| Admission age (days) | 0 (0–0) |
| Male gender | 2,353 (60%) |
| Race/ethnicity |  |
| Asian | 64 (2%) |
| Black | 527 (13%) |
| Hispanic | 683 (17%) |
| White | 2,053 (53%) |
| Chromosomal abnormality | 475 (12%) |
| Public/governmental insurance | 2,091 (54%) |
| Household income ($1,000) | 40 (33-51) |
| Hospital charges ($1,000) | 703 (380-1,373) |

**Table S3. Characteristics of all neonates with HLHS.** Values expressed as median (IQR) or n (%) as appropriate.
